# Purification and characterization of cysteine protease from germinating cotyledons of horse gram

**DOI:** 10.1186/1471-2091-10-28

**Published:** 2009-11-17

**Authors:** Rajeswari Jinka, Vadde Ramakrishna, Sridhar K Rao, Ramakrishna P Rao

**Affiliations:** 1 Center for Cellular and Molecular Biology, Uppal Road, Hyderabad - 500 007, India; 2 Department of Biotechnology and Bioinformatics, Yogi Vemana University, Kadapa - 516 003. A.P. India; 3 Department of Biochemistry, Sri Krishnadevaraya University, Anantapur - 515 003. Andhra Pradesh, India

## Abstract

**Background:**

Proteolytic enzymes play central role in the biochemical mechanism of germination and intricately involved in many aspects of plant physiology and development. To study the mechanism of protein mobilization, undertaken the task of purifying and characterizing proteases, which occur transiently in germinating seeds of horse gram.

**Results:**

Cysteine protease (CPRHG) was purified to homogeneity with 118 fold by four step procedure comprising Crude extract, (NH4)2SO4 fractionation, DEAE-Cellulose and CM-sephacel chromatography from the 2 day germinating cotyledons of horse gram (*Macrotyloma uniflorum *(Lam.) Verdc.). CPRHG is a monomer with molecular mass of 30 k Da, was determined by SDS-PAGE and gel filtration. The purified enzyme on IEF showed two isoforms having pI values of 5.85 and 6.1. CPRHG composed of high content of aspartic acid, glutamic acid and serine. The enzyme activity was completely inhibited by pCMB, iodoacetate and DEPC indicating cysteine and histidine residues at the active site. However, on addition of sulfhydryl reagents (cysteine, dithiothreitol, glutathione and beta-ME) reverse the strong inhibition by pCMB. The enzyme is fairly stable toward pH and temperature. Immunoblot analysis shows that the enzyme synthesized as zymogen (preproenzyme with 81 kDa) and processed to a 40 kDa proenzyme which was further degraded to give 30 kDa active enzyme.

**Conclusion:**

It appears that the newly synthesized protease is inactive, and activation takes place during germination. CPRHG has a broad substrate specificity and stability in pH, temperature, etc. therefore, this protease may turn out to be an efficient choice for the pharmaceutical, medicinal, food, and biotechnology industry.

## Background

Proteolytic enzymes are multifunctional enzymes that have many physiological functions in plants and animals including germination, senescence, apoptosis, complement activation, inflammation process etc. and also having commercial importance in food, leather and textile industry. Commercially they are extremely important as more than 60% of the total enzyme market is made up of proteases; they are isolated from plants, animals, bacteria and fungi. Proteolytic enzymes from the plant sources have received special attention because of their broad substrate specificity as well active in wide range of pH, temperature, and in presence of organic compounds as well as other additives [1,2]. Search for valuable proteases with distinct specificity is always a continuous challenge for varied industrial applications.

The mobilization of seed storage proteins represents one of the most important post-germination events in the growth and development of seedling. Proteolytic enzymes play central role in the biochemical mechanism of germination and intricately involved in many aspects of plant physiology and development [2,3]. Numerous reports including our previous data supported the proteases are responsible for protein degradation. The legume seeds contain albumin and globulin storage proteins; act as amino acid reserves which are mobilized to nourish the seedling. Globulins belong to the vicillin and legumin family these are degraded by endoproteases particularly cysteine proteinase. In horse gram, during germination we observed the disappearance of high molecular weight (HMW) polypeptides of globulins and appearance of a new 25 kDa polypeptide [4-9]. To study the mechanism of protein mobilization process, many have undertaken the task of purifying and characterizing a variety of proteases, some of which occur only transiently in germinating seeds [2,10,11]. With renewed interest, there has been proliferation of reports in the last decade concerning purification and characterization of these proteases from germinating leguminous and non-leguminous seeds [12-16]. Exploration for existence of valuable proteases as well as understanding the appropriate physiological role of such proteases in plants is still an open area of investigation.

Horse gram (*Macrotyloma uniflorum *(Lam.) Verdc.) is one of the lesser known, unexploited legume of the tropics and subtropics grown under dry-land agriculture. In our previous studies the pattern of mobilization of seed storage proteins and activation of proteolytic enzymes (endoprotease, carboxypeptidase and leucine aminopeptidase) were investigated in germinating horse gram seeds [7,17]. In the present investigation we report the purification and biochemical characteristics of an endoprotease from the germinating seedlings of horse gram. It is shown that this protease is a cysteine protease (CPRHG) hitherto not known in the genus *Macrotyloma *with novel cleavage specificities.

## Results

### Purification of protease

**Figure 1:**
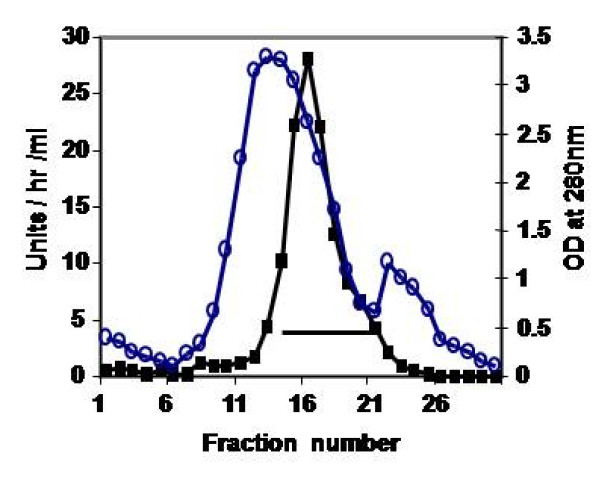
**Fractionation of enzyme on DEAE-Cellulose column**: The enzyme fraction obtained from ammonium sulfate precipitation (30-60%) was loaded on to DEAE-Cellulose column equilibrated with equilibrating buffer (0.01 M Tris- HCl buffer, pH 7.0, containing 2 mM β-ME) at a flow rate of 40 ml/hr and 5 ml fractions were collected and assayed for enzyme activity as described in materials and methods. The indicated fractions (14-20) were pooled for further processing.

**Figure 2:**
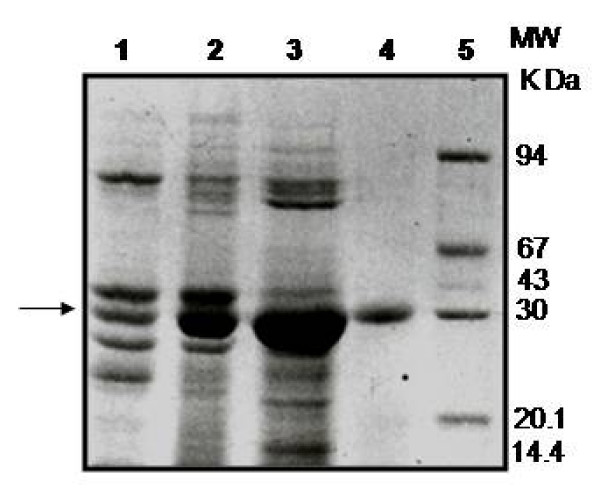
**SDS-PAGE analysis of the fractions from each step of endoprotease (EP-HG) purification from the cotyledons of germinating horse gram seeds and seedlings**. Conc. of protein loaded on each well in gel is 100 ug. Lane 1. Crude extract, Lane 2. Protein from 30-60% saturated ammonium sulfate fraction. Lane 3. Pooled fractions (14-20) after DEAE-Cellulose chromatography. Lane 4. Unbound fraction from CM-Sephacel column. Lane 5. Molecular weight markers consisting of Phosphorylase b (94 k Da), BSA (67 k Da), ovalbumin (43 k Da), carbonic anhydrase (30 k Da), soybean trypsin inhibitor (20.1 k Da) and α-lactalbumin (14.4 k Da).

The cotyledons of day 2 germinating horse gram seeds were used for isolation of endoprotease, since the activity was maximal at this point during the four-day period of germination [7]. Maximum yield of protease activity observed with 0.05 M Tris-HCl buffer pH 7.2, containing 2 mM of β-ME among other buffers (acetate, borate, phosphate buffers) used. Change in pH, alterations in molarity or omission of β-ME in the extraction buffer substantially lowered the yield of the activity (data not shown). The protease activity was measured by using the chromogenic substrate, azocasein. The results of the four step purification of CPRHG were summarized in Table 1. The CPRHG was initially precipitated with (30% - 60%) ammonium sulphate and dialyzed and subjected to DEAE cellulose column. The bound enzyme was eluted with a linear gradient of KCl (0 - 0.5 M). The fractions [14-20] contained 40% of enzyme activity with specific activity of 4.80 were pooled and concentrated by ammonium sulphate (Fig. 1). The final efficient step of purification is the fractionation on CM-Sephacel. Under provided conditions, 12% of the loaded enzyme was not adsorbed to the column and eluted, that was found to be homogeneous on 10% SDS-PAGE (Fig. 2).

**Table 1:** Summary of the purification of CPRHG from germinating horse gram seeds

Purification step	Total Protein (mg)	Total activity (Units/hr)	Specific activity (Units/mg protein)	Yield (%) Enzyme activity	Purification fold
Crude extract	9405.00	1578.00	0.168	100.00	1.00
Ammonium sulfate precipitation (30 - 60%)	2713.00	1187.00	0.437	75.22	2.44
DEAE-cellulose	130.80	628.00	4.801	40.00	28.27
CM-Sephacel	3.65	72.58	19.88	4.59	118.36

(Averages of triplicate analysis of each step)

### Homogeneity and size of the purified enzyme

**Figure 3:**
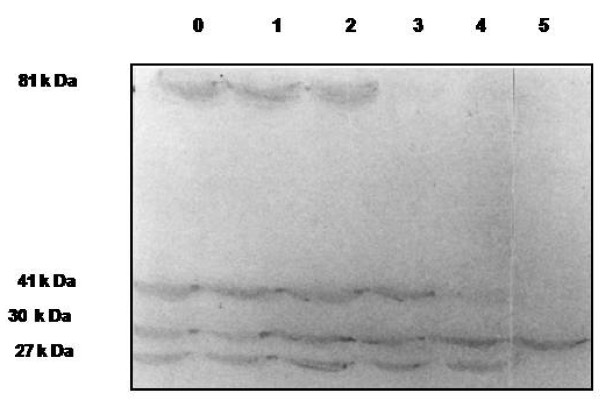
**Western blot analysis of crude extracts and purified CPRHG against anti- CPRHG polyclonal antibodies**: 8% SDS-PAGE, 100 μg loaded on the each lane of the gel. Lanes 0-4: Cotyledon extracts at days 0,1,2,3 and 4, respectively, from germinating horse gram seedlings. Lane 5: Purified CPRHG.

**Figure 4:**
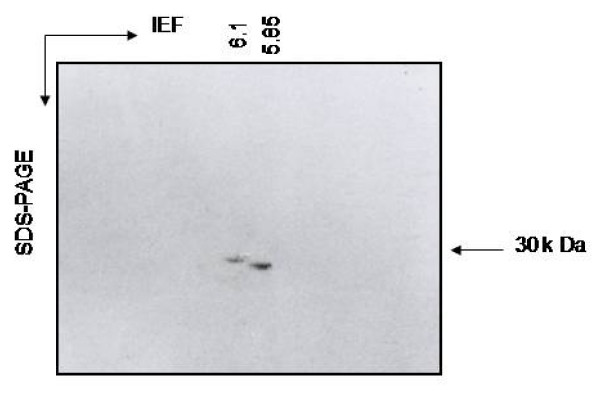
**Two dimensional (2D) gel electrophoresis of CPRHG**: The first dimension isoelectric focusing was performed in a tube gel using wide range of ampholytes (pH 3-10). Second dimension was on 10% SDS-PAGE and was stained with coomassie brilliant blue.

The purified CPRHG was found to be homogeneous by the detection of a single polypeptide by SDS-PAGE which is further supported by single precipitin band on immunodiffusion of the crude extract (result not shown). Immunoblot analysis of the purified CPRHG also conform the monoreactivity of the antiserum (Fig 3 lane 5). The size of the purified CPRHG was estimated to be 30.2 K Da on Sephadex G-100 column and the same was also supported by SDS-PAGE (Fig. 2). However, on isoelectrofocussing (IEF) two isoforms were observed with different pI values of 5.85 and 6.1 (Fig 4).

### Amino acid composition

The amino acid analysis of CPRHG was shown relatively fewer numbers of Histidines, sulfur containing amino acids (Cys and Met) and higher number of aspartic acid (Asp), glutamic acid (Glu) and serine (Table 2).

**Table 2:** Amino acid composition of purified CPRHG

Aminoacid	No. of residues
Asx	33
Thr	20
Ser	30
Glx	25
Pro	16
Gly	19
Ala	24
Cys	2
Val	19

### Effect of pH, temperature, time and storage stability

**Figure 5:**
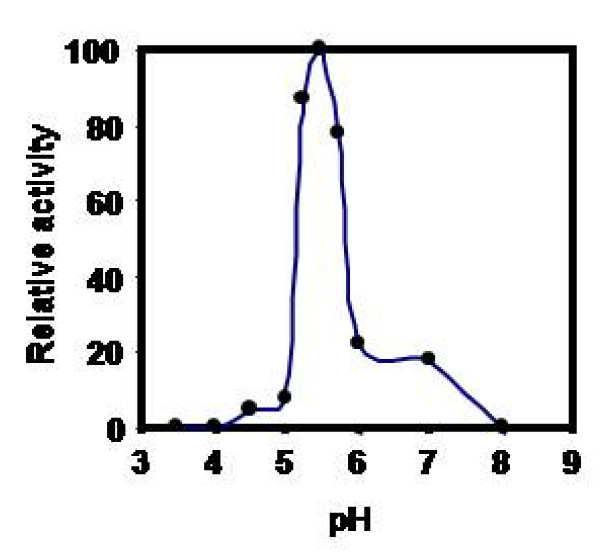
**Effect of pH on the activity of CPRHG**. Azocasein is the substrate for enzyme assay.

**Figure 6:**
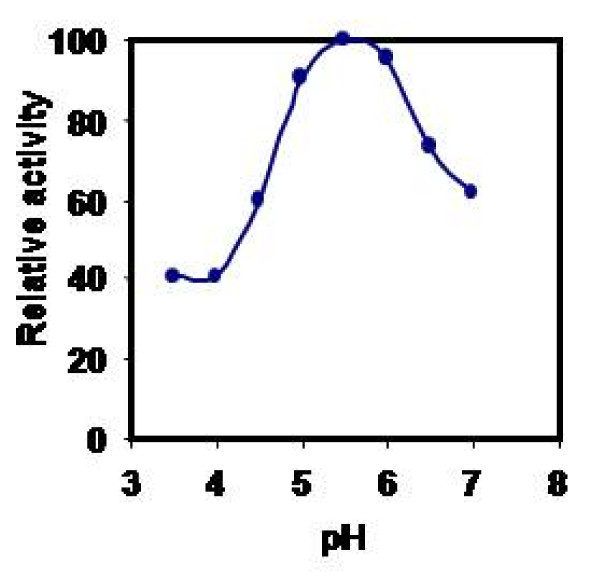
**PH stability of CPRHG**. Azocasein is the substrate for enzyme assay.

**Figure 7:**
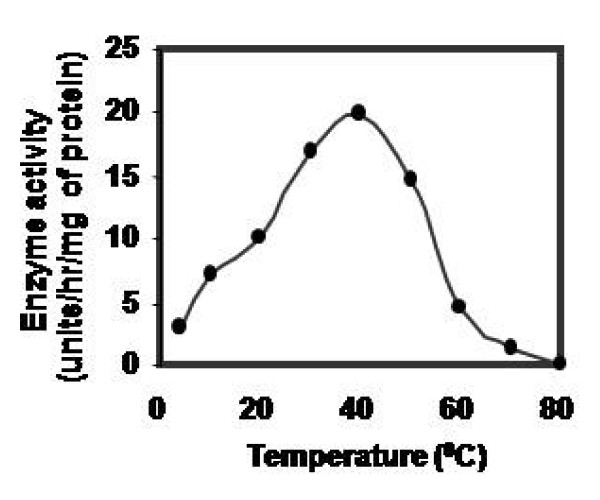
**Temperature- activity profile of CPRHG**. Azocasein is the substrate for enzyme assay.

**Figure 8:**
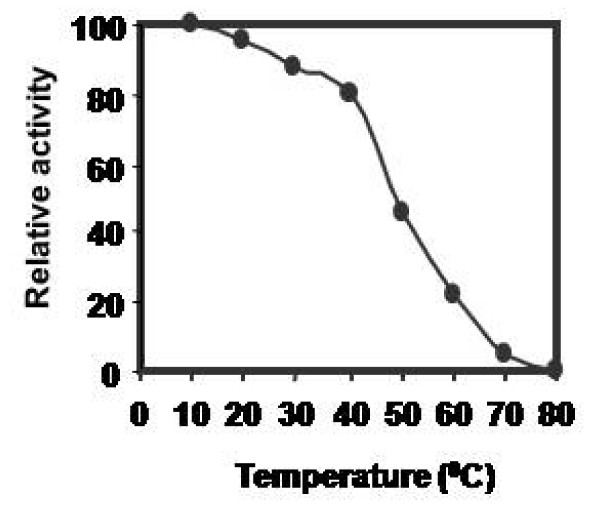
**Thermal stability of CPRHG**. Azocasein is the substrate for enzyme assay. The activity was expressed as per cent of activity at 10°C.

**Figure 9:**
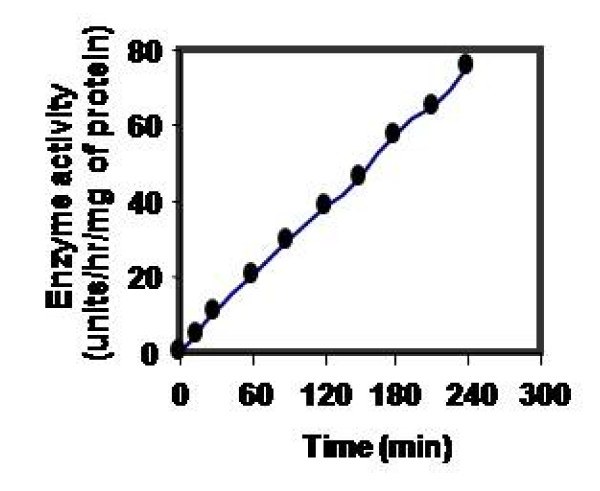
**Effect of time on reactivity of CPRHG**. Azocasein is the substrate for enzyme assay.

Effect of pH on CPRHG was carried out using azocasein as substrate and found the enzyme was active at acidic pH optimum, 5.5 with azocasein (Fig 5), 4.0 with hemoglobin and 5.2 with gelatin and BSA (data not shown). The activity was stable in the pH range 5.0- 6.0 (Fig 6) and also observed gradual loss of activity outside this pH range. However, the temperature vs activity profile showed an increased activity with temperature and exhibited maximal activity at 40°C (Fig 7). The enzyme was fairly stable at 40°C (Fig 8) and also exhibits linear proteolytic activity up to 4 h at this temperature (Fig 9).

### *In vitro *digestion of endogenous and exogenous proteins

**Figure 10:**
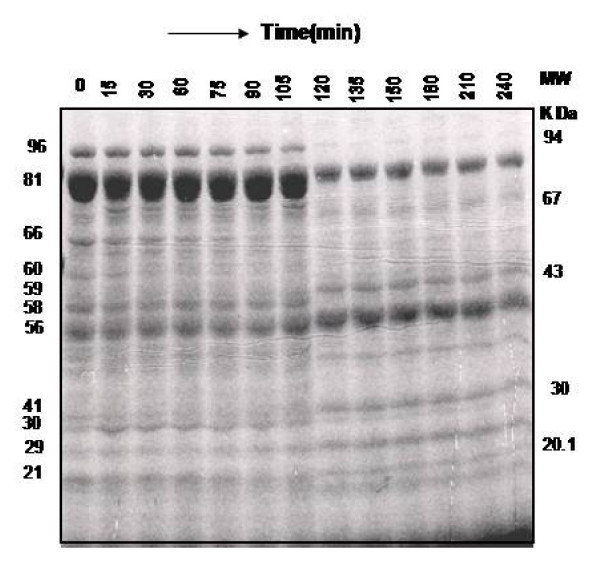
***In vitro *digestion of native horse gram seed proteins by CPRHG**. 100 μg of digestion mixture was loaded on the each lane of the 8% SDS-PAGE gel.

Table 3 showed the comparative hydrolytic activity of the purified CPRHG with endogenous (seed proteins) and exogenous proteins (BSA, casein, gelatin and hemoglobin). Seed protein degradation was remarkably higher than the gelatin, casein, BSA and hemoglobin. The enzyme was unable to cleave Leu-p-nitroanilide and CBZ-L-phenylalanine. Digestion of endogenous proteins by CPR HG on time course study through SDS-PAGE shows no appreciable degradation within first 90 min. However, the disappearance of polypeptides of 96, 81, 66, 60, 58 and 21 k Da and appearance of new polypeptides of 73 and 59 k Da and intensification of 56 and 29 kDa bands were observed on SDS-PAGE with increasing digestion time (Fig 10).

**Table 3:** Hydrolysis of protein substrates by purified CPRHG

Substrate	Conc. of substrate	pH	Reaction rate (mmoles of amino acids released/hr/mg)
Horse gram seed proteins	1%	5.5	612.34
BSA	1%	5.2	126.60
Casein	1%	5.5	132.16
Gelatin	1%	5.5	220.80
Hemoglobin	1%	4.0	106.04
CBZ-L- Phenyl alanine	2 mM	5.5	Nil
Leu-p-nitroanilide	2 mM	5.6	Nil

The rate of hydrolysis is expressed as m moles of amino acids released/h/mg enzyme

### Effect of metal ions, selective inhibitors and sulfhydryl reagents on CPRHG

The protease activity was clearly inhibited at lower concentrations of Zn^2+^, Hg^2+ ^and Cu^2+ ^(0.5 mM). However, metal ions like Ni^2+^, Co^2+^and Pb^2+ ^were shown an inhibition on above 1 mM. Mn^2+ ^at higher concentrations (above 1 mM) stimulated the activity. Metal chelating agents such as 1, 10 phenanthroline and EDTA had no effect (Table 4 &5). The CPRHG activity was neither inhibited by aspartyl modifying reagent (pepstatin) nor by serine modifying reagents (DIPF, soybean trypsin inhibitor, eserine, PMSF and aprotinin). However, the activity was completely inhibited by sulfhydryl modifying reagents (pCMB and iodoacetamide). We also observed the complete inhibition by histidyl modifying reagent (DEPC). These results suggested that the CPRHG is a cysteine protease, with cysteine and histidine residues at the active site (Table 5). Addition of sulfhydryl reagents (cysteine, dithiothreitol, glutathione and β-ME) reverses the strong inhibition by pCMB (Table 6 &7).

**Table 4:** Effect of various metal ions on purified CPRHG

	Relative activity (%)				
	
Reagent	Conc. of reagents (mM)				
	
	0.5	1.0	2.0	5.0	10
NiCl_2_	81	86	68	63	57
CoCl_2_	73	76	63	57	56
Cupric acetate	19	13	11	7	4
Manganese chloride	101	101	111	136	158
Lead acetate	89	90	89	64	34
Zinc acetate	58	35	20	8	2
Magnesium Chloride	116	102	102	102	95
Mercuric Chloride	20	15	7	3	0
Control	100	100	100	100	100

The hydrolyzing activity is expressed as per cent of control (19.73 units/h/mg enzyme)

**Table 5:** Effect of various inhibitors on CPRHG activity

Inhibitor	**Conc**.	Relative activity (%)
pCMB	10 mM	0
NEM	100 mM	61
Iodoacetamide	100 mM	8
DTNB	0.2%	40
DIPF	10 mM	93
STI	0.05%	99
Eserine	0.01%	96
pMSF	100 mM	81
Bestatin	0.1 mg/ml	92
Aprotinin	0.001%	96
Pepstatin	0.01%	88
DEPC	10 mM	0
EDTA	10 mM	95
1, 10-Phenanthroline	10 mM	99
Control	-	100

The hydrolyzing activity is expressed as per cent of control (19.73 units/h/mg enzyme)

**Table 6:** Effect of various Sulfhydryl reagents on CPRHG

Conc. of reagents (mM)	Relative activity (%)			
	
	Glutathione	Cysteine	β-ME	Dithiothretol
0.5	100	106	107	112
1.0	121	114	120	**116**
2.0	115	110	115	**131**
5.0	119	105	98	**127**
10.0	172	103	92	**106**
Control	100	100	100	**100**

The hydrolyzing activity is expressed as per cent of control (19.73 units/h/mg enzyme)

**Table 7:** Reactivation of pCMB treated CPRHG by sulfhydryl reagents

Substance added	Concentration (mM)	Relative activity (%)
Control	-	100
pCMB	10	0
pCMB + β-ME	5 10	53 77
pCMB + DTT	5 10	73 75
pCMB +Glutathione	5 10	85 104
pCMB + Cysteine	5 10	71 83

The hydrolyzing activity is expressed as per cent of control (19.73 units/h/mg enzyme)

### Immunoblotting of CPR HG development

The polyclonal anti-CPRHG could recognize 81, 41, 30 and 27 k Da polypeptides from crude extract of germinating cotyledons. Presence of HMW 81 k Da protein in the first 2 days of germination and disappeared by day 3. The 41 k Da polypeptide was noticed in the extracts from days 0-3. The intensity of 30 k Da endoprotease (CPR HG) in the cotyledons of germinating seeds increased up to day 2 and declined later. Similar profile was also noticed for 27 k Da polypeptides (Fig 3). These results revealed that the enzyme CPRHG synthesized as zymogen (preproenzyme with 81 K Da) and processed to a 40 K Da proenzyme and then to the 30 K Da active enzyme which is further degraded to a 27 K Da product.

## Discussion

Maximum endoprotease activity was observed in day 2 cotyledons of horse gram seeds during 4 day germination period and also shown pH optima in acidic region suggesting that the enzyme is located in the vacuoles [3,7,10,17,18]. The role(s) of specific proteases in seed protein degradation is unclearly understood, indeed, the overall role has been focus for direct investigation. We made an attempt to purify the endoprotease (CPRHG) from the cotyledons of horse gram and studied its properties and physiological role in the degradation of storage proteins. The purification procedure yielded an essentially homogeneous preparation with an over all recovery of 4.59% and 118 fold purification. The final recovery of CPRHG (4.59%) was similar to that of other plant cysteine proteases i.e., vicilin peptidohydrolase (8.0%) from mung bean seedlings [19] and GA3- induced protease (3.38%) from barley aleurone layers [20], 15% acidic protease from germinating winged bean [12] and 12% from Indian beans [16]. The specific activity of the purified aspartic protease was 0.64 U/mg with a recovery of 20% and existed as a single form from *Ficus racemosa *[15].

CPRHG appeared to be monomeric protein with molecular weight of 30 k Da (Fig 2) similar to other cysteine proteases isolated from germinating *Vicia sativa *[5], soyabean [21], barley [22] and wheat [23]. However, the molecular weights of cysteine proteases isolated from *Vigna mungo *seeds varied between 20-30 k Da [24] and in barley 30- 37 k Da [25,26]. Purified CPRHG was found to contain two isoforms with iso-electric points, pI 5.85 and 6.1 (Fig 4) as that of aleurain, isolated from aleurone cells, with two isoforms of pI values 6.0 and 6.1 and purified thiol protease from barley also had multiple forms including EP-A and EP-B [20].

CPRHG exhibits acidic pH optima by showing higher activity at 5.5. Discrepancy in pH optima was noticed for various protein substrates used in the assay, since a given substrate will have numerous ionizable groups with similar pKa values. The pH optimum obtained with a protein substrate reflects more about the pH - mediated susceptibility of the substrate to proteolysis than about the influence of pH on the protease as a catalyst. A similar susceptibility to pH had been described for endopeptidase from *Phaseolous vulgaris *[27], barley [20] and wheat [28]. Since the purified CPRHG was found to be stable in mildly acidic pH range 5.0 - 6.0, the enzyme may be localized *in vivo *in protein bodies like in other legume seeds [6,7,10-13,29]. The enzyme (CPRHG) exhibited surprisingly high temperature optima at 40°C and the catalytic reaction was linear with time for at least 4 hr at this temperature, which reflects resistance to autolysis and the results were correlated to the proteases of lentil seeds [30] and barley [20,22].

CPRHG showed a high degree of specificity towards the natural substrates i.e., seed proteins from horse gram seeds and had completely an endoproteolytic activity, which suggested an important role in the mobilization of seed proteins during germination. *In vitro *digestion of seed proteins by purified CPRHG led to the gradual disappearance of high molecular weight polypeptides. An aspartic endoprotease from wheat also exhibited the similar endoproteolytic activity with no exopeptidase activity [28]. Studies with *Phaseolous vulgaris *[31], *Phaseolus mungo *[32], mung bean [33], *Glycine max *[13,34], wheat [35] and winged bean [12] also indicated the role of cysteine endoprotease in the mobilization of stored proteins.

We have employed various inhibitors specific to each class of proteinases (metallo-, asp-, serine and cys-) in order to investigate the amino acid residue(s) contributing to the active site of the enzyme. Inhibition of the enzyme activity by heavy metal ions and inhibitors, sulfhydryl blocking reagents, and the reactivation of pCMB - treated enzyme by the addition of sulfhydryl reagents clearly established the thiol nature of the enzyme. However, the partial inhibition by other sulfhydryl blocking reagents suggests the involvement of other residue(s) also in enzyme activity. The strong inhibition of CPRHG by DEPC suggested the involvement of histidine at the active site along with cysteine as proposed for papain [36]. However, thiol proteases purified from winged bean [12] and *Phaseolous vulgaris *[37] did not belong to the papain family. Generally all thiol proteases have three conserved pairs of cysteine residues in their mature domain. However CPRHG contained only one pair of cysteine residues (Table 2). This data suggested that CPRHG showed homology to other proteases, such as EP-C1 of *Phaseolous vulgaris *[37] and EP-A of barley [20].

Temporal changes in the levels of CPRHG in the cotyledons of germinating horse gram seeds were observed by immunoblotting. In addition to 30 k Da protein (CPRHG), the other three polypeptides with 81, 41 and 27 k Da was also cross-reacted with the antiserum. These results are similar to the development of endoprotease activity in germinating horse gram seeds, where increased development from day 0 to day 2 and decreased further [7]. Information obtained from NBRF protein sequence data bank and translated Gen Bank database indicated that several cysteine proteases are produced as zymogens [26]. The polypeptide (CPRHG) with ≥ 30 k Da in the immunoblot appeared corresponding to the putative enzyme and the 81 and 41 k Da polypeptides to the corresponding prepro- and pro-enzymes. The 27 k Da polypeptide probably originated from the 30 k Da protease. The endoproteses in the seeds of *Vigna mungo *[38], castor beans [39] and barley [20,40] were found to be synthesized as proenzymes and processed to the active forms during germination.

## Conclusion

The temporal variations in CPRHG activity, its acidic pH optimum and its ability to degrade native seed storage proteins preferentially fulfilled the criteria laid down for a protease to be involved in seed storage protein degradation during germination. It appears that the newly synthesized protease is inactive, and activation takes place during germination. The reported enzyme has broad substrate specificity and stability in pH, temperature, etc., therefore, this protease may turn out to be an efficient choice in pharmaceutical, medicinal, food, and biotechnology industry.

## Methods

### Chemicals

Sephadex G-100, CM-Sephacel, LMW markers were procured from Pharmacia Fine Chemicals, Uppsala, Sweden and DEAE-cellulose, aprotinin, p-chloromercuribenzoate (PCMB), N-ethylmaleimide (NEM), phenylmethylsulfonylfluoride (PMSF), iodoacetamide, Soybean trypsin inhibitor, eserine, diisopropylflourophosphate (DIFP), bestatin and 1,10- phenanthroline, diethylpyrocarbonate (DEPC), CBZ-L-phenyl alanine and L-Leu-p-nitroanilide were purchased from Sigma Chemical company, USA. All other chemicals, unless otherwise specified, were of analytical grade and purchased from Specrochem (India) and Qualigens (India).

### Plant Material

Horse gram (*Macrotyloma uniflorum *(Lam.) Verdc.) seeds were procured from Agricultural Farm of Andhra Pradesh Agricultural University, Rekulakunta, Anantapur, Andhra Pradesh, India.

### Germination conditions

Horse gram seeds were surface sterilized with 0.1% HgCl_2 _solution for 5 min, washed repeatedly with sterile water and soaked in 10 volumes of water for four hours. The imbibed seeds were set to germinate at room temperature (30 ± 2°C) at 12 h dark and 12 h light cycle for four days in sterile petri dishes lined with four layers of filter paper. Sterile conditions were maintained by including 20 ppm of streptomycin sulphate in the incubation medium (water). The cotyledons harvested for two days were used for the isolation of CPR HG.

### Preparation of the enzyme extract

Cotyledons were ground thoroughly in a mortar adding four volumes of chilled 0.05 M Tris-HCl buffer, pH 7.2, containing 2 mM β-ME. The extract was filtered through four layers of cheesecloth and the filtrate was centrifuged at 10000 g for 15 min at 4°C. The supernatant was used for isolation of the enzyme. All separation procedures were carried out at 4°C, unless otherwise stated.

### Purification of CPRHG from germinating horse gram cotyledons

The crude enzyme (endoproteinase) extract was prepared as described above from 100 g of cotyledons (day 2) was subjected to ammonium sulfate precipitation. The precipitate obtained between 30% - 60% ammonium sulfate saturation was collected and dissolved in minimal amount of extraction buffer and extensively dialyzed against 0.01 M Tris- HCl buffer, pH 7.2 containing 2 mM β-ME at 4°C (1:200 volume ratio with 2 h change upto 12 h). The dialysate was loaded on DEAE-cellulose column equilibrated with 0.01 M Tris- HCl buffer, pH 7.0 containing 2 mM β-ME. The bound proteins were eluted with a linear gradient of increasing ionic strength of potassium chloride (0.5 M) in equilibration buffer and 5 ml fractions were collected. The fractions (14-20) with higher enzymatic activity were pooled and brought to 60% saturation with pulverized ammonium sulfate at 4°C and centrifuged. The pellet was dissolved in minimal volume of 0.02 M sodium acetate buffer, pH 5.5 containing 2 mM β-ME and dialyzed against the same buffer at 4 °C for 12 hr. The dialysate was loaded on CM-Sephacel column equilibrated with 0.02 M sodium acetate buffer, pH 5.5 containing 2 mM β-ME. The unbound fractions were collected and concentrated by lyophilization and stored at -20°C. Criteria of purity at each stage was checked by 10% SDS-PAGE.

### Assay of endoprotease

Endoprotease (EP-HG) activity was measured by using chromogenic substrate, azocasein, following the method described by Sarath et al [41] with slight modifications. 0.25 ml of 1% azocasein (prepared in 0.02 M sodium acetate buffer, pH 5.5 containing 2 mM β-ME) was mixed with 0.15 ml of enzyme extract or 150 μg of purified enzyme (1 mg/ml) and incubated at 40°C for 1 hr. The reaction was arrested by adding 1.2 ml of 10% TCA and mixed thoroughly. The contents were allowed to stand for 15 min and centrifuged for 5 min at 3000 rpm. 1.2 ml of the supernatant was transferred to a tube containing 1.4 ml of 1 M NaOH, mixed and the absorbance was read at 440 nm against the reagent blank. One unit of protease activity was defined as the amount of the enzyme required to produce an absorbance change of 1.0 in 1 cm cuvette under the conditions of the assay.

### Determination of amino acid composition

The protein was hydrolyzed with 6N HCl for 24 h at 110°C in an evacuated sealed tube. The hydrolysate was filtered through Whatman No.1 filter paper and the filtrate was evaporated to dryness in a flash evaporator and the amino acid composition was analyzed on model 119 CL Beckman amino acid analyzer. Cysteine residues were measured by performic acid method [42].

### Iso-electric focusing

IEF and 2D electrophoresis of purified enzyme was carried out according to the method of O' Farrel [43].

### Determination of MW of CPRHG

The molecular weight of the purified protease was determined by Sephadex G-100 gel filtration (2.4 × 132 cm column, flow rate 2 ml/min) and also through SDS-PAGE [44].

### Effect of pH and temperature on enzyme

The effect of pH on enzyme activity was determined by carrying experiment at different pH using sodium acetate buffer (pH 3.5 - 5.5), sodium phosphate buffer (pH 5.6- 7.0) and Tris-HCl buffer (pH 7.2 - 9.2). The enzyme activity was assayed as described. The effect of temperature on the enzyme activity was also determined at different temperatures ranging from 10-80°C for 1 hr in 0.02 M sodium acetate buffer pH 5.5, containing 2 mM β-ME using the same assay as described.

### Storage stability of enzyme

The storage stability of the of the purified enzyme (CPRHG) was investigated by storing the enzyme at different temperatures, -10°C, 4°C and 30°C and its stability for a week was tested by withdrawing an aliquots of the enzyme at different intervals of time and was assayed for the enzyme activity.

### Time course study

The purified CPRHG and azocasein were taken as described earlier and incubated at 40°C for 4 hr. Aliquots were withdrawn from incubation mixture at different intervals of time from 0 - 4 hrs and the azo compound liberated was measured as described.

### Antisera preparation

Polyclonal antibodies against the CPRHG were raised in rabbits by injecting 100 μg of the purified protein. Antibodies were used for immunoinhibition, immunodiffusion and immunoblot to check their specificity (44).

### Western blot analysis for developmental pattern of CPRHG in the cotyledons of germinating horse gram seedlings

Extracts of the cotyledons from germinating seeds harvested at daily intervals for 4 days were subjected to SDS-PAGE and the proteins were transferred on to a nitrocellulose paper. The proteins on nitrocellulose paper were treated with primary antibody (anti-CPRHG) and successively with secondary antibody (Ig-alkaline phosphotase). The bands were visualized by using NBT and BCIP in Tris HCl buffer, pH 9.0, containing 10 mM MgCl_2 _and 100 mM NaCl [44].

### Effect of metal ions, selected inhibitors and sulfhydryl reagents

150 μg of CPRHG in 0.02 M sodium acetate buffer pH 5.5 was preincubated with 10 μl of different metal ions, selected inhibitors and sulfhydryl reagents at varied concentrations for 1 hr at room temperature and the assay was initiated by the addition of azocasein and the activity assayed as described.

### Effect of -SH reagents on pCMB treated EP- HG

pCMB (10 mM) treated protease was reincubated prior to the assay with sulfhydryl reagent (5 mM and 10 mM) for 1 hr at room temperature and the activity was assayed as described. The proteolytic activity was compared with the control experiment containing all the components except the SH-reagent.

### Hydrolysis of endogenous and exogenous substrates

The relative digestibility of endogenous and exogenous proteins by the purified CPRHG was measured by incubating 1 ml of 1% protein (dry seed protein, BSA, casein, hemoglobin, gelatin) prepared in an appropriate buffer mixed with 0.15 ml of the purified CPRHG (1 mg/ml) and incubated at 40°C for 1 hr and the amino acids released were estimated by ninhydrin method [45]. Presence of exopeptidase activity was checked by using L-Leu-p-nitroanilide and CBZ-L-phenylalanine as described by Chrispeels and Boulter [46] and Sarath et al. [41], respectively.

### *In vitro *digestion of seed proteins (SDS-PAGE analysis)

Suitable volumes of dry seed protein extract (1%) and CPRHG (1 mg/ml) were incubated as described, at various intervals aliquots were withdrawn from the digestion mixture, mixed with 2× sample buffer, boiled for 3 min and subjected to SDS-PAGE.

## Abbreviations used

CPRHG: Cysteine Protease of Horse gram; DEAE: Diethyl aminoethylcellulose; CM: Carboxymethyl; pCMB: p-chloro mercuribenzoate; NEM: N-ethylmaleimide; PMSF: phenyl methylsulfonylfluoride; DIFP: diisopropylflourophosphate; DEPC: diethyl pyrocarbonate; CBZ: carboxybenzyl; NBRF: National Biomedical Research Foundation; EP: Endopeptidase; EDTA: Ethylene diamine tetra aceticacid; DTT: Dithiothreitol; STI: Soyabean trypsin inhibitor.

## Authors' contributions

RJ designed and carried out the entire study of purification and characterization. VRK helped in drafting the manuscript. KSR helped in designing the experiments. PRR conceived of the study and participated in its design and coordination and helped to draft the manuscript. All authors read and approved the final manuscript.

## References

[B1] Uhlig H Industrial Enzymes and their Applications 1998 John Wiley & Sons, Inc., New York 146 151

[B2] Schaller A A cut above the rest: the regulatory function of plant proteases Planta 2004 220 183 197 10.1007/s00425-004-1407-2 15517349

[B3] Muntz K Belozersky MA Dunaevsky YE Schlereth A Tiedemann J Stored proteinases and the initiation of storage protein mobilization in seeds during germination and seedling growth Journal of Experimental Botany 2001 52 1741 1752 10.1093/jexbot/52.362.1741 11520862

[B4] Rotari V Senyuk V Horstmann C Jivotovskaja AV Vaintraub IA Proteinase A-like enzyme from germinated kidney bean seeds. Its action on phaseolin and vicilin Physiologia Plantarum 1997 100 171 177 10.1111/j.1399-3054.1997.tb03469.x

[B5] Tiedemann I Neubohn J Muntz K Different functions of vicillin and legumin are reflected in the histopattern of globulin mobilization during germination of vetch (*Vicia sativa *L.) Planta 2001 211 1 12 10.1007/s004250000259 10923698

[B6] He F Huang F Wilson KA Wilson AT Protein storage vacuole acidification as a control of storage protein mobilization in soybeans Journal of Experimental Botany 2007 58 1059 1070 10.1093/jxb/erl267 17229757

[B7] Rajeswari J Ramakrishna Rao P Storage protein degradation in germinating horse gram seeds Ind J Plant Physiol 2002 7 314 320

[B8] Ramakrishna V Ramakrishna Rao P Storage protein degradation in germinating Indian bean (*Dolichos lablab L. var lignosus*) seeds Seed Science and Technology 2006 34 161 168

[B9] Zakharov A Carchilan M Stepurina T Rotari V Wilson K Vaintraub IA A comparative study of the role of the major proteinases of germinated common bean (*Phaseolus vulgaris *L.) and soybean (*Glycine max *(L.) Merrill) seeds in the degradation of their storage proteins Journal of Experimental Botany 2004 55 406 410 10.1093/jxb/erh247 15333645

[B10] Shutov AD Vaintraub IA Degradation of storage proteins in germinating seeds Phytochemistry 1987 26 1557 1566 10.1016/S0031-9422(00)82245-1

[B11] Jones BL Endoproteases of barley and malt J Cereal science 2005 42 139 156 10.1016/j.jcs.2005.03.007

[B12] Usha R Singh M Proteases of germinating winged bean (*Psophocarpous tetragonolobus) *seeds: purification and characterization of an acidic protease Biochem J 1996 313 423 429 8573074 10.1042/bj3130423 PMC1216925

[B13] Asano M Suzuki S Kawai M Miwa T Shibai H Characterization of novel cysteine proteases from germinating cotyledons of soybean *[Glycine max (L.) Merrill]* J Biochem 1999 126 2 296 301 10423520 10.1093/oxfordjournals.jbchem.a022448

[B14] Beilinson V Moskalenko OV Livingstone DS Reverdatto SV Jung R Nielsen NC Two subtilisin-like proteases from soybean Physiol Plant 2002 115 4 585 597 10.1034/j.1399-3054.2002.1150413.x 12121465

[B15] Devaraj KB Lalitha R Gowda V Prakash V An unusual thermostable aspartic protease from the latex of *Ficus racemosa *(L.) Phytochemistry 2008 69 647 655 10.1016/j.phytochem.2007.09.003 17936863

[B16] Ramakrishna V Ramakrishna Rao P Purification of acidic protease from the cotyledons of germinating Indian bean (*Dolichos lalab L*. var lignosus) seeds African Journal of Biotechnology 2005 4 7 703 706

[B17] Karunagaran D Ramakrishna Rao P Axial control of protease development in cotyledons of horse gram *(Macrotyloma uniform Lam*.) seeds during germination Indian J Plant Physiol 1990 33 232 238

[B18] Jiang L Rogers JC Integral membrane protein sorting to vacuoles in plant cells: evidence for two pathways Journal of Cell Biology 1998 143 1183 1199 10.1083/jcb.143.5.1183 9832548 PMC2133091

[B19] Baumgartner B Chrispeels MJ Purification and Characterization of Vicilin Peptidohydrolase, the Major Endopeptidase in the Cotyledons of Mung-Bean Seedlings Euro J Biochem 1977 77 223 233 10.1111/j.1432-1033.1977.tb11661.x 891531

[B20] Koehler S T-HD Ho A major gibberellic acid induced barley aleurone cystein proteinase which digests hordein. Purification and characterization Plant Physiol 1990 94 251 258 10.1104/pp.94.1.251 16667694 PMC1077218

[B21] Seo S Tan-Wilson A Wilson KA Protease C2, a cysteine endopeptidase involved in the continuing mobilization of soybean beta conglycinin seed proteins Biochim Biophys Acta 2001 1545 192 206 11342045 10.1016/s0167-4838(00)00277-6

[B22] Jones BL Poulle M A proteinase from germinated barley II. Hydrolytic specificity of a 30 K Da cysteine proteinase from green malt Plant Physiol 1990 94 1062 1070 10.1104/pp.94.3.1062 16667797 PMC1077342

[B23] Bottari A Capocchi A Galleschi L Japova A Saviozzi F Asparagine endopeptidase during maturation and germination of drum wheat Physiologia Plantarum 1996 97 475 480 10.1111/j.1399-3054.1996.tb00506.x

[B24] Mitsuhashi W Koshiba T Minamikawa T Seperation and characterization of two endopeptidases from cotyledons of germinating *Vigna mungo *seeds Plant Physiol 1986 80 628 634 10.1104/pp.80.3.628 16664675 PMC1075173

[B25] Holwerda BC Rogers JC Purification and characterization of aleurain Plant Physiol 1992 99 848 855 10.1104/pp.99.3.848 16669011 PMC1080555

[B26] Wrobel R Jones BL Appearance of endoproteolytic enzymes during the germination of barley Plant Physiol 1992 100 1508 1516 10.1104/pp.100.3.1508 16653151 PMC1075813

[B27] Boylon MJ Sussex IM Purification of an endopeptidase involved with storage protein degradation in *Phaseolous vulgaris *L. cotyledons Planta 1987 170 343 352 10.1007/BF00395026 24232964

[B28] Belozersky MA Sarbakanova ST Dunaevsky YE Aspartic proteinase from wheat seeds: isolation, properties and action on gliadins Planta 1989 177 321 326 10.1007/BF00403589 24212424

[B29] Liu X Zhang Z Barnaby N Wilson KA Tan-Wilson A Soybean subtilisin-like protease involved in initiating storage protein degradation Seed Science Research 2001 11 55 68

[B30] Guerra H Nicolas G Purification and characterization of two proteolytic enzymes in the cotyledons of germinating lentils Rev Esp Fisiol 1983 39 283 289 6361931

[B31] Nielson SS Liener IE Degradation of the major storage proteins of *Phaseolous vulgaris *during germination- role of endogenous protease and protease inhibitors Plant Physiol 1984 74 494 508 10.1104/pp.74.3.494 16663450 PMC1066714

[B32] Minamikawa T Hydrolytic enzyme activities and degradation of storage compounds in cotyledons of germinating *Phaseolous mungo *seeds Bot Mag Tokyo 1979 92 1 12 10.1007/BF02488296

[B33] Harris N Chrispeels MJ Histochemical and biochemical observations on storage protein metabolism and protein body autolysis in cotyledons of germinating mung beans Plant Physiol 1975 56 292 299 10.1104/pp.56.2.292 16659290 PMC541807

[B34] Bond HM Bowles DJ Characterization of soybean endopeptidase activity using exogenous and endogenous substrates Plant Physiol 1983 72 345 350 10.1104/pp.72.2.345 16663004 PMC1066235

[B35] Taylor RM Cuming AC Purification of an endoproteinase that digests the wheat 'Em' proteins in vitro and determination of its cleavage sites FEBS Lett 1993 331 76 80 10.1016/0014-5793(93)80300-J 8405415

[B36] Dunn MJ Protein purification methods: A practical approach 1989 IRL Press, NY, USA

[B37] Csoma C Polgar L Proteinase from germinating bean cotyledons. Evidence for involvement of a thiol group in catalysis Biochem J 1984 222 769 776 6385962 10.1042/bj2220769 PMC1144241

[B38] Mitsuhashi W Minamikawa T Synthesis and post translational activation of sulfhydryl- endoprotease in cotyledons of germinating *Vigna mungo *seeds Plant Physiol 1989 89 274 279 10.1104/pp.89.1.274 16666526 PMC1055831

[B39] Schmid M Simpson D Kalousek F Gietl C A cystein endoprotease with a C-terminal KDEL motif isolated feom castor bean endosperm is a marker enzyme for the ricinosome, a putative lytic compartment Planta 1998 206 466 475 10.1007/s004250050423 9763713

[B40] Martilla S Porali I T-HD Ho Mikkonen A Expression of the 30 KD cystein endoprotease B in germinating barley seeds Cell Biol Int 1993 17 205 212 10.1006/cbir.1993.1056

[B41] Sarath G Dela motte RS Wagner FW "Proteolytic Enzyme. A practical approach" 1989 IRL Press, Oxford, England Inc

[B42] Hirs CHW Methods Enzymol 1967 11 59 62 full_text

[B43] O'Farrell PH High resolution Two- Dimensional Electrophoresis of proteins J Biol Chem 1975 250 4007 4021 236308 PMC2874754

[B44] Sambrook J Fritsch EF Maniatis T "Molecular Cloning", Laboratory manual 1989 Second Cold Spring Harbor Laboratory Press, New York, USA

[B45] Rosen R A modified ninhydrin colorimetric analysis for amino acids Arch Biochem Biophys 1957 67 10 15 10.1016/0003-9861(57)90241-2 13412116

[B46] Chrispeels MJ Boulter D Control of storage protein metabolism in the cotyledons of germinating mung bean: role of endopeptidase Plant Physiol 1975 55 1031 1037 10.1104/pp.55.6.1031 16659204 PMC541760

